# ABI3BP promotes renal aging through Klotho-mediated ferroptosis

**DOI:** 10.1186/s12967-024-05300-w

**Published:** 2024-05-29

**Authors:** Ren Ji, Lin Wei, Yuxin Zan, Xiao Li, Shinan Ma, Liming Ma, Xiju He, Li Wang, Yan Ding

**Affiliations:** 1 Hubei Key Laboratory of Embryonic Stem Cell Research, Hubei Provincial Clinical Research Center for Umbilical Cord Blood Hematopoietic Stem Cells, Taihe Hospital, Hubei University of Medicine, Shiyan, 442000 Hubei China; 2 Center for Clinical Laboratories, The Affiliated Hospital of Guizhou Medical University, Guiyang, China; 3 Urology Department, Taihe Hospital, Hubei University of Medicine, Shiyan, 442000 Hubei China

**Keywords:** ABI3BP, Renal aging, Klotho, Ferroptosis

## Abstract

The aging process of the kidneys is accompanied with several structural diseases. Abnormal fiber formation disrupts the balance of kidney structure and function, causing to end-stage renal disease and subsequent renal failure. Despite this, the precise mechanism underlying renal damage in aging remains elusive. In this study, ABI3BP gene knockout mice were used to investigate the role of ABI3BP in renal aging induced by irradiation. The results revealed a significant increase in ABI3BP expression in HK2 cells and kidney tissue of aging mice, with ABI3BP gene knockout demonstrating a mitigating effect on radiation-induced cell aging. Furthermore, the study observed a marked decrease in Klotho levels and an increase in ferroptosis in renal tissue and HK2 cells following irradiation. Notably, ABI3BP gene knockout not only elevated Klotho expression but also reduced ferroptosis levels. A significant negative correlation between ABI3BP and Klotho was established. Further experiments demonstrated that Klotho knockdown alleviated the aging inhibition caused by ABI3BP downregulation. This study identifies the upregulation of ABI3BP in aged renal tubular epithelial cells, indicating a role in promoting ferroptosis and inducing renal aging by inhibiting Klotho expression.

## Background

As the elderly population continues to grow, so does life expectancy. Advancing age triggers significant changes in various organ systems. The kidney, with its high energy metabolism, emerges as a particularly sensitive target organ susceptible to age-related tissue damage [1]. Throughout the aging process, the kidney undergoes a series of abnormal changes in structure and function, including increased renal vascular resistance, reduced renal plasma flow, and elevated glomerular filtration fraction. Morphological changes include cortical mass loss, tubular atrophy, interstitial fibrosis, and glomerulosclerosis [2]. Understanding the mechanisms behind renal aging is crucial, given its significance in preventing age-related renal fibrosis.

Cellular aging, a hallmark of aging in general, is marked by a significant rise in iron ion levels. This increase, nearly tenfold compared to normal cells, can be inhibited by the antioxidant N-tert-butylhydroxylamine. This suggests that iron accumulation triggers oxidative stress and cellular dysfunction, ultimately leading to cellular senescence [3]. Dysfunctional iron metabolism has been linked to elevated iron levels and ferroptosis, a form of cell death. Studies further suggest a connection between ferroptosis and various kidney diseases: targeted iron-induced cell death may provide therapeutic benefit in acute or chronic kidney injury, diabetic nephropathy, and renal cell carcinoma [4–7]. Moreover, drugs that counteract lipid peroxidation or remove iron from cells have been shown to extend lifespan in nematodes [8]. While these findings suggest a link between ferroptosis and aging, the specific role of ferroptosis in the context of kidney function and HK-2 cells remains to be elucidated.

The extracellular matrix provide a supportive environment for many types of cells and ensure the mechanical stability of cells. It modulates substance transport, cell growth, migration, aging and differentiation [9]. Studies have shown that extracellular matrix proteins may be important signs of aging [10]. ABI3BP, a recently discovered extracellular matrix protein, has been linked to tumor development in numerous studies [11, 12]. Knocking down ABI3BP expression has been shown to inhibit tumor growth by affecting tumor cell proliferation, viability, migration, and invasion [13]. Latini et al. [14] suggested that ABI3BP might suppress tumor growth through the p21 pathway, regulating the cell cycle. Furthermore, ABI3BP has been reported to promote cellular senescence in a p53-dependent manner, which plays a crucial role in replicative senescence and may be a factor in tumor development. However, the potential role of ABI3BP in promoting cellular aging via ferroptosis remains unclear. This study investigates the involvement of the extracellular matrix protein ABI3BP in renal aging through the lens of ferroptosis.

## Materials and methods

### Animals and ethics statement

ABI3BP knockout (ABI3BP^−/−^) mice were purchased from the model animal Platform of the Experimental Animal Center at Huazhong Agricultural University (China, Hubei). Mouse gene identification primers are shown in Table S1. C57BL/6 mice were purchased from Hubei University of Medicine (China, Hubei). All mice were housed in specific pathogen-free (SPF) conditions at the experimental animal center of Hubei Medical College. The animal rearing room maintained a temperature of 22℃ with a light cycle of 12 h light and 12 h dark. All animal experiments were performed on healthy male mice, aged 8w old (20 ± 2 g). The use of all mice adhered to the guiding principles of the Animal Ethics Committee of Hubei Medical College, following the ethical policies and procedures approved by the Welfare Ethics Committee of Hubei University of Medicine (2021-S-033).

### Cell culture

HK-2 cells were seeded onto 6-well plates during their logarithmic growth phase. Upon reaching 70% confluency, the cells were irradiated with 8 Gy X-rays and subsequently cultured in complete medium for 24 h.

### Western blot

30 μg of protein was separated on a 12% SDS-PAGE gel. Following electrophoresis, the proteins were transferred to a PVDF membrane. The membranes were then sealed with 5% skimmed milk powder for 2 h at room temperature, washed with TBST buffer, and incubated with corresponding primary antibodies overnight at 4℃. These included rabbit anti-ABI3BP antibody (bioss, BS-6506r, 1:500), rabbit Anti- KL Polyclonal antibody (Proteintech, 28100-1-AP, 1:500), rabbit Anti- GPX4 Polyclonal antibody (Proteintech, 30388-1-AP, 1:500), rabbit Anti- NRF2 Polyclonal antibody (Proteintech, 16396-1-AP, 1:500), rabbit Anti- Acsl4 Polyclonal antibody (Proteintech, 22401-1-AP, 1:500), and GAPDH mouse monoclonal antibody (Beyotime, AF0006, 1:1000). After three washes with TBST buffer, the membranes were incubated with horseradish peroxidase-labeled secondary antibody at room temperature for 2 h. Following another three washes with TBST buffer, protein bands were visualized using BeyoECL Plus (Beyotime, P0018S) and the Bio-Rad-Image-Lab system.

### Quantitative RT-PCR

Total RNA was extracted from HK-2 cells using TRIzol reagent. The RNA was reverse-transcribed to cDNA using the HiScript IV RT SuperMix reagent by qPCR (+ gDNA wiper) (Vazyme, R423) following the manufacturer’s instructions. The cDNA was then mixed with the Taq Pro Universal SYBR qPCR Master Mix (Vazyme, Q712) and specific primers for quantitative RT-PCR. Primers for GAPDH (Homo) were: forward, 5′-CCGGGAAACTGTGGCGTGATGG-3′; reverse, 5′-AGGTGGAGGAGTGGGTGTCGCTGTT-3′. The primers for ABI3BP (Homo) were: forward, 5′-AGGCCAAACCTCAAAGTCCAC-3′; reverse, 5′-GGTGATACATTGCTGCCATATCC-3′. The primers for Klotho (Homo) were: forward, 5′-CCCTAAGCTCTCACTGGATCA-3', reverse, 5′-GGCAAACCAACCTAGTACAAAGT-3′.

### Radiation-induced aging

HK-2 cells in logarithmic growth phase were inoculated in 6-well plates. When the density reached 70%, they were irradiated with 8 Gy X-rays, and then cultured in complete medium for 24 h.

Wild-type (WT) and ABI3BP knockout (ABI3BP^−/−^) 8-week-old male C57BL/6 mice were randomly divided into groups: WT-Irradiation group and ABI3BP^−/−^-Irradiation group, with 10 mice in each group. Mice in these groups were irradiated 3 times with 5 Gy X-rays at an interval of 21 days, while those in the control group were not irradiated with the same other variables.

### Immunofluorescence staining

Cell or tissue samples were fixed with 4% paraformaldehyde at room temperature for 10 min, permeabilized with 0.1% Triton X-100 for 10 min, and incubated with corresponding primary antibody at 4℃ overnight. Primary antibodies included rabbit anti- Histone H2A.X antibody (Proteintech, 10856-1-AP, 1:200), rabbit anti- P53 Polyclonal antibody (Proteintech, 10442-1-AP, 1:200), rabbit anti- P21 Polyclonal antibody (Proteintech, 10355-1-AP, 1:200), rabbit anti-Klotho Polyclonal antibody (Proteintech, 28100-1-AP, 1:200), rabbit anti- GPX4 Polyclonal antibody (Proteintech, 30388-1-AP, 1:200), and rabbit anti- Nrf2 Polyclonal antibody (Proteintech, 16396-1-AP, 1:200). Next, the samples were incubated with the corresponding fluorescent secondary antibody at room temperature for 1 h, and DAPI staining reagent for 10 min. Finally, an anti-fluorescence quenching tablet was used to seal the film, and images were captured using a fluorescence microscope (Olympus, Japan).

### Immunohistochemistry (IHC)

4 μm paraffin-embedded mouse kidney tissue sections were used for immunohistochemical staining, following our previously established method [15]. The details of the antibodies used were as follows: rabbit anti-Klotho Polyclonal antibody (Proteintech, 28100-1-AP, 1:200 dilution), rabbit anti-GPX4 Polyclonal antibody (Proteintech, 30388-1-AP, 1:200 dilution), and rabbit anti-Nrf2 Polyclonal antibody (Proteintech, 16396-1-AP, 1:200 dilution), rabbit anti-ABI3BP antibody (Bioss, BS-6506r, 1:400 dilution). IHC slices were scanned and statistically analyzed by Olympus microscope and Image J, respectively.

### SA-β-Gal staining

The Sa-β-Gal Staining Kit (Beyotime, C0602) was used to detect cell senescence. HK2 cells were washed with PBS, and fixed at room temperature for 15 min with a fixing solution. The cells were then stained with dyeing solution (prepared according to instruction B) and incubated overnight in a carbon dioxide-free incubator at 37 ℃. After incubation, pictures were taken with a microscope (Olympus, Japan).

### Co-IP

HK-2 cells were lysed on ice with RIPA lysis buffer for 30 min, then centrifuged at 12,000 *g* for 20 min. The obtained supernatant was mixed with Klotho or IgG antibody, and incubated at 4℃ overnight. The protein-antibody mixture was further incubated with protein A/G-agarose beads (Santa Cruz Biotechnology) at 4 ℃ for 4–6 h. Finally, protein samples were collected for western blot.

### Plasmid construction and transfection

The Klotho CRISPR/Cas9 vector provided by Weizhen was used. HEK293T cells were transfected with three plasmid systems (pMD2G, pSPAX2, Sg-Klotho) and packaged as lentivirus. 48 h after the lentivirus infection of HK-2 cells, 2.5 μg/mL puromycin was added to screen the successfully expressed cells. Western blot was used to confirm the knock-out efficiency.

### Biochemical index detection

The levels of creatinine (C011-2-1), urea nitrogen (C013-2-1) and malondialdehyde (MDA, A003-1-2) in serum were measured according to the operation instructions of the corresponding detection kits (Nanjing Jiancheng Bioengineering Institute).

### CCK-8 cell viability assay

HK-2 cells were plated in 96-well plates with 5000 cells per well. Before the test, 10 μL CCK-8 reagent (Beyotime, C0042) was added to 100 μL culture medium, and incubated at 37℃ for 1 h. Cell activity was evaluated by measuring the absorbance at 450 nm using an enzyme-labeled instrument.

### Intracellular ferrous ion fluorescence probe detection and Prussian blue dyeing

HK-2 cells were washed with PBS for three times. Then, 1 mol/L FerroOrange working solution (FUSHENBIO, FS1349) was added and cultured in a 5% CO_2_ incubator at 37℃ for 30 min. The specific protocols for Prussian blue staining of mice kidney tissue sections were conducted following instructions on the kit (Solarbio, G1422). Images were taken with a microscope (Olympus, Japan).

### Lipid peroxidation MDA detect

Following the manufacturer’s instructions (Beyotime, Cat. No. S0131S), HK-2 cells were lysed and centrifuged at 10,000–12,000×*g* for 10 min. The supernatant was then collected for protein concentration determination, and used to conduct lipid peroxidation assay according to the kit protocol.

### Statistical analysis

Statistical analysis of the data were performed using GraphPad Prism8.0 (GraphPad Inc, USA). Numerical data were presented as “mean ± SD (standard deviation)”. Inter-group differences were analyzed using a two-tailed Student’s t-test. A P-value < 0.05 was considered statistically significant.

## Results

### ABI3BP expression level is up-regulated in aging kidney tissue induced by radiation

The role of ABI3BP, an extracellular matrix protein, in kidney disease remains unclear. This study investigated ABI3BP expression in aging kidney tissue induced by radiation. Analysis of The Human Proteins atlas database revealed a significant up-regulation of ABI3BP in kidney tissues of elderly individuals (Fig. S1A). Moreover, using aging-related kidney diseases animal models (GSE145053, GSE126182, GSE79443, GSE189377, and GSE216376), we observed a consistent up-regulation of ABI3BP expression (Fig. S1B-F).

**Fig. 1:**
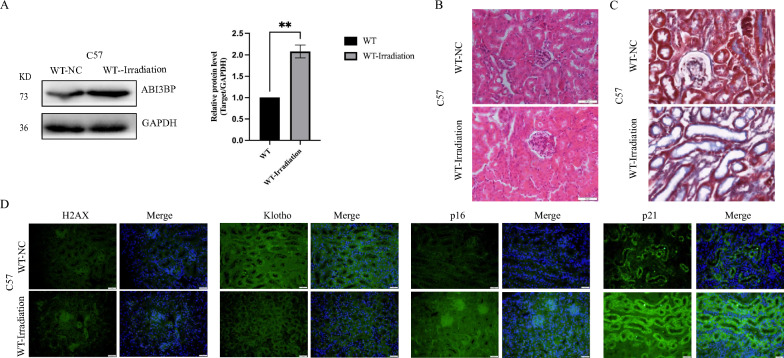
ABI3BP upregulation in aging kidney tissue induced by radiation. **A** Western Blot analysis of ABI3BP expression in radiation-induced aging mouse kidney tissue (n = 3). **B** HE staining for renal tissue morphology and Masson staining for renal fibrosis in WT irradiated mice (scale bar = 50 μm, n = 3). **C** Tissue immunofluorescence detecting H2AX, Klotho, P16, and P21 protein expression in WT-Irradiation mice kidney tissue (scale bar = 20 μm, n = 3). n = 3 biologically independent repeats. Two-sided Student’s t-test, data shown as mean ± SD. *P < 0.05, **P < 0.01

Osipov et al. [16], established that X-ray irradiation accelerates mouse aging. Therefore, in our study, mice were randomly divided into a control group and a radiation-induced aging group. Following three instances of 5 Gy irradiation every 21 days, we assessed the relevant indices of kidney tissue aging. Western blot results illustrated a clear up-regulation of ABI3BP expression in the kidneys of irradiated mice (Fig. 1A). HE staining detected renal tubular atrophy and Masson staining also indicated aggravated renal fibrosis in irradiated mice (Fig. 1B). Concurrently, levels of P16, P21, and γ-H2AX increased, while Klotho decreased (Fig. 1C). Additionally, a unilateral ureteral obstruction (UUO) model of kidney injury in mice demonstrated evident hydrops and fibrosis in the UUO group, with significantly increased blood urea nitrogen and elevated ABI3BP expression in both blood and kidney tissues (Fig. S2). These findings suggest that ABI3BP expression is up-regulated in aging-related kidney diseases, potentially acting as a negative factor for kidney health.

### ABI3BP expression level is up-regulated in aging HK-2 cells induced by radiation

**Fig. 2:**
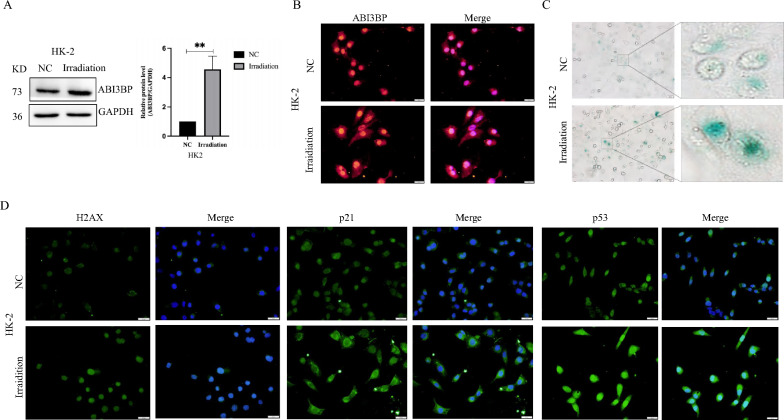
ABI3BP up-regulation in aging HK-2 cells induced by radiation. **A** Western Blot analysis of ABI3BP expression in radiation-induced aging HK-2 cells (n = 3). **B** Cell immunofluorescence showing ABI3BP protein expression in irradiated HK-2 cells (scale bar = 20 μm, n = 3). **C** SA-β-Gal staining of control and irradiated HK-2 cells (scale bar = 20 μm, n = 3). **D** Immunofluorescence detecting H2AX, P21, and P53 proteins in irradiated HK-2 cells (scale bar = 20 μm, n = 3). n = 3 biologically independent repeats. Two-sided Student’s t-test, data shown are mean ± SD. *P < 0.05, **P < 0.01

Cell aging is a fundamental process with intricate connections to various age-related diseases. Using single-cell data analysis databases for healthy adult human and mouse kidneys, we observed high ABI3BP expression in proximal tubule cells (Fig. S3A-B). Given the high energy demand of renal proximal tubular epithelial cells, any disruptions in their energy homeostasis are associated with the progression of renal diseases. X-ray irradiation serves as an inducer of cellular aging. After 24 h of irradiation, we assessed the aging status of HK-2 cells by measuring the fluorescence intensity of P21, P53, and H2AX. Immunoblotting (Fig. 2A) and cellular immunofluorescence (Fig. 2B) demonstrated a significant up-regulation of ABI3BP expression in aging HK-2 cells. SA-β-Gal staining revealed a notable increase in the positive rate of WT-irradiated HK-2 cells (Fig. 2C), accompanied by elevated levels of P21, P53, and γ-H2AX, as indicated by cellular immunofluorescence (Fig. 2D). Collectively, these findings strongly suggest that ABI3BP acts as an unfavorable factor for in the aging process of HK-2 cells.

### ABI3BP knockout alleviates radiation-induced renal aging in mice

**Fig. 3:**
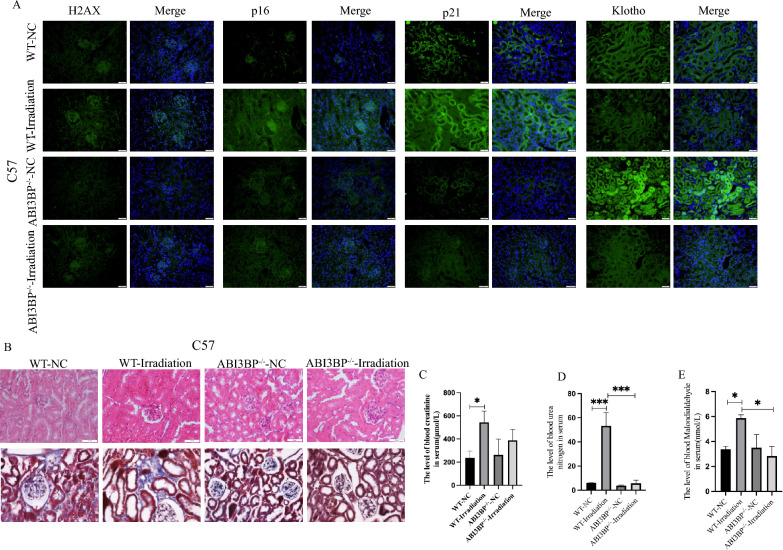
ABI3BP knockout alleviates kidney radiation-induced kidney aging in mice. **A** Tissue immunofluorescence of γ-H2AX, Klotho, P16, and P21 proteins in WT-NC, WT-Irradiation, ABI3BP^−/−^NC, ABI3BP^−/−^ irradiation (n = 3) mice (scale bar = 20 μm). **B** HE staining for renal tissue morphology and Masson staining for renal fibrosis in four groups of mice (n = 3, scale bar = 50 μm). **C**–**E** Detection of serum creatinine (**C**), urea nitrogen (**D**), and MDA (**E**) in WT-NC, WT-Irradiation, ABI3BP^−/−^ NC, and ABI3BP.^−/−^ irradiation mice (n = 4). n = 3 biologically independent repeats. Two-sided Student’s t-test, data presented as mean ± SD. *P < 0.05, **P < 0.01, ***P < 0.001

To investigate the specific effect of ABI3BP on renal aging, we constructed ABI3BP gene knockout mice (ABI3BP^−/−^, Fig. S4A-C) and used radiation to induce aging. Surprisingly, compared to the wild-type group, ABI3BP^−/−^ mice exhibited a noticeable improvement in radiation-induced renal aging. The levels of γ-H2AX, P16, and P21 were reduced, while Klotho levels increased (Fig. 3A). Additionally, ABI3BP^−/−^ provided protection against renal tubular atrophy and tubulointerstitial fibrosis caused by irradiation (Fig. 3B). Considering the crucial role of renal proximal tubule cells in maintaining electrolyte and acid–base balance, we assessed relevant renal function indexes. The ABI3BP^−/−^ group showed a significant decrease in serum creatinine (Fig. 3C), urea nitrogen (Fig. 3D), and MDA levels (Fig. 3E), further supporting the protective effect of ABI3BP^−/−^ group.

Verification through the UUO model demonstrated a significant reduction in hydronephrosis and fibrosis levels in the ABI3BP^−/−^ group (Fig. S4D-G). These findings indicate that ABI3BP knockout either protected or reversed renal function damage caused by harmful factors.

### ABI3BP knockout suppresses radiation-induced ferroptosis, alleviating aging

**Fig. 4:**
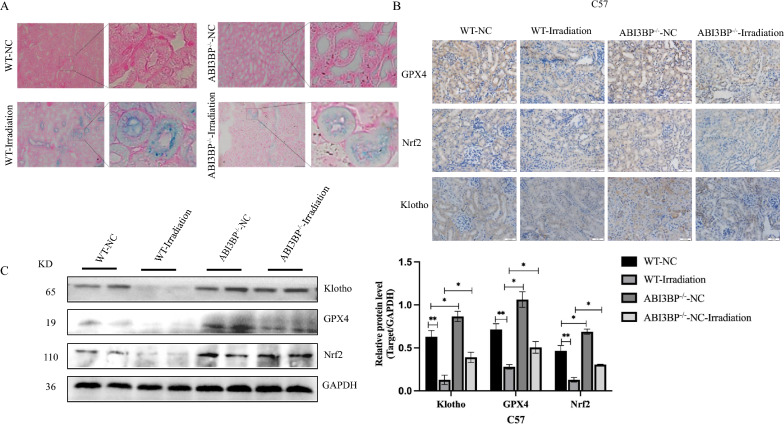
ABI3BP knock out alleviates ferroptosis generation in radiation-induced mice kidneys. **A** Detection of Fe^3+^ content in mouse kidney tissue using Ruslane staining. **B** Immunohistochemistry of GPX4, Nrf2, and Klotho protein expression in four groups of mice (n = 3, scale bar = 50 μm). **C** Western Blot quantifying protein levels of Nrf2, GPX4, and Klotho in four groups (n = 3). Data presented as mean ± SD. n = 3 biologically independent repeats. Two-sided Student’s t-test, data shown are mean ± SD. *P < 0.05, **P < 0.01

Recent studies highlight the close relationship between ferroptosis and cell senescence. Iron ion modulation plays a pivotal role in ferroptosis, and assessing iron ion content can indicate its occurrence. Prussian blue staining of mouse kidney tissue revealed that irradiation increased iron ions, a response mitigated by ABI3BP knockout (Fig. 4A). Immunohistochemistry and western blot analysis of ferroptosis-related indicators GPX4, Nrf2, and the renal aging indicators Klotho confirmed their significant decrease after irradiation (Fig. 4B, C). ABI3BP knockout enhanced the stability of GPX4, Nrf2, and Klotho proteins, consequently inhibiting ferroptosis.

**Fig. 5:**
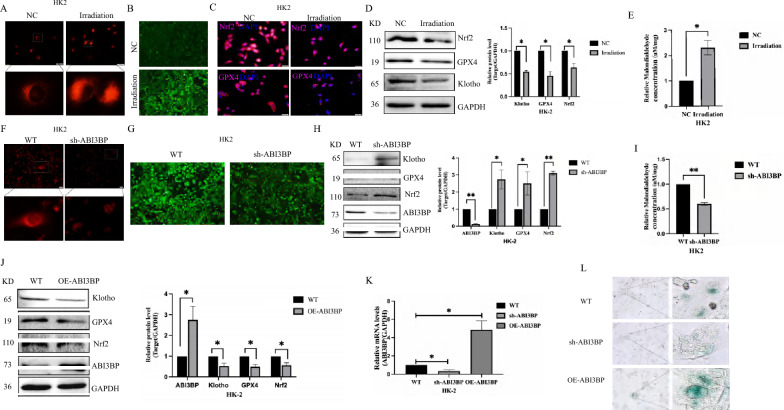
ABI3BP promotes cell senescence by increasing ferroptosis production. **A** Detection of Fe^2+^ content in aged HK-2 cells using a ferrous ion fluorescence probe (scale bar = 20 μm, n = 3). **B** Increased ROS content in aging HK-2 cells (n = 3). **C** Immunofluorescence of Nrf2 and GPX4 protein expression in aging HK-2 cells (scale bar = 20 μm, n = 3). **D** Western Blot showing down-regulation of Nrf2, GPX4, and Klotho protein levels in aging HK-2 cells (n = 3). **E** MDA level of HK-2 cells after irradiation (n = 3). **F** Detection of Fe^2+^ content in HK-2 cells using a ferrous ion fluorescence probe (n = 3). **G** Decreased ROS production in HK-2 cells (n = 3). **H** Western Blot showing protein levels of Nrf2, GPX4, and Klotho in HK-2 cells (n = 3). **I** Malondialdehyde level of different HK-2 cells (n = 3). **J** the protein levels of Nrf2, GPX4 and Klotho in HK-2 cells detected by western blot (n = 3). **K** Quantitative RT-PCR showing the knockout and overexpression efficiency of ABI3BP in HK-2 cells (n = 3). **L** The number of SA-β-Gal staining positive cells in indicated groups (scale bar = 50 μm, n = 3). n = 3 biologically independent repeats. Two-sided Student’s t-test, data shown are mean ± SD. *P < 0.05, **P < 0.01

To validate the in vivo results, we investigated ABI3BP’s effect on ferroptosis in vitro. Consistent with the in vivo findings, irradiation-induced increased ferroptosis. In cellular aging, we employed a ferrous ion fluorescence probe to assess the Fe^2+^ content in irradiated HK-2 cells (Fig. 5A). The accumulation of iron within cells serves as a hallmark of ferroptosis, and excessive Fe^2+^ mediates the generation of ROS (Fig. 5B). Our evaluation revealed a significant decrease in the expression of GPX4 and Nrf2 after irradiation, as demonstrated by cellular immunofluorescence and immunoblotting (Fig. 5C, D), confirming the involvement of GPX4 in the ferroptotic process. Importantly, sh-ABI3BP inhibited ferroptosis and alleviated cellular aging, as evidenced by reduced Fe^2+^ content (Fig. 5E), decreased ROS production (Fig. 5F), and the up-regulation of key proteins GPX4 and Nrf2 (Fig. 5G). Simultaneously, we established ABI3BP overexpressing HK-2 cells, confirming successful overexpression. Following ABI3BP overexpression, a significant decrease in the expressions of GPX4 and Nrf2 was observed (Fig. 5H). Moreover, the proportion of SA-β-gal positive senescent cells increased upon ABI3BP overexpression and decreased after sh-ABI3BP treatment (Fig. 5I).

We further constructed HK-2 cells with overexpression of ABI3BP, which was successfully confirmed (Fig. 5J). Following ABI3BP overexpression, we observed a significant decrease in the expression levels of GPX4 and Nrf2 (Fig. 5J). This finding was corroborated by quantitative RT-PCR results, which revealed statistically significant differences in ABI3BP knockdown and overexpression at the RNA level (Fig. 5K). Moreover, the proportion of SA-β-gal positive senescent cells increased upon ABI3BP overexpression and decreased with sh-ABI3BP treatment (Fig. 5L). These findings collectively suggest that ABI3BP can promote intracellular iron-dependent cell death (ferroptosis) and contribute to cellular senescence.

### ABI3BP promotes cell senescence by regulating Klotho to induce ferroptosis

**Fig. 6:**
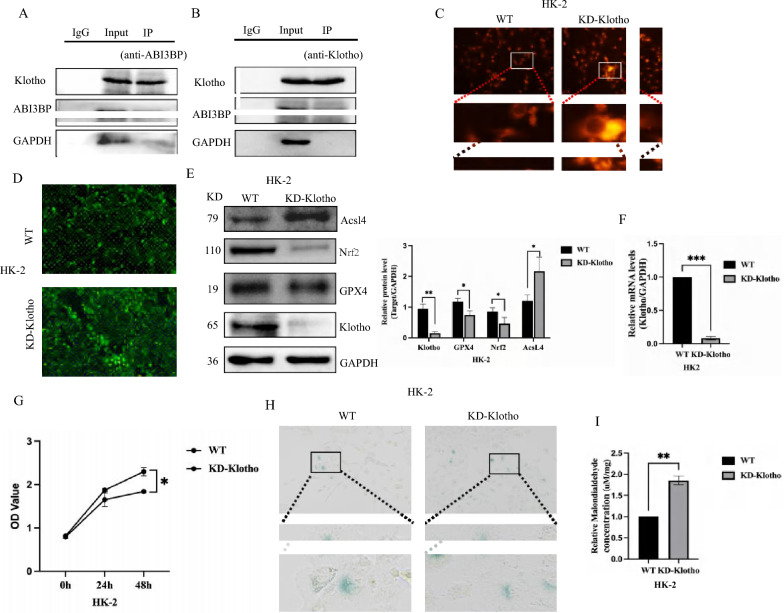
ABI3BP promotes cell senescence by regulating Klotho to induce ferroptosis. **A**, **B** Co-IP analysis revealed an interaction between ABI3BP and Klotho, but with no interaction observed between ABI3BP and GAPDH or Klotho and GAPDH. **C** Significantly increased Fe^2+^ content in HK-2 cells following the detection of KD-Klotho using a ferrous ion fluorescence probe (n = 3). **D** KD-Klotho, the ROS production of HK-2 cells increased significantly. **E** Western Blot analysis detected protein levels of Nrf2, GPX4, and Acsl4 after KD-Klotho treatment in HK-2 cells (n = 3). **F** Quantitative RT-PCR detection the efficiency of KD-Klotho in HK-2 cells (n = 3). **G** CCK8 essay assessed the proliferation ability of HK-2 cells with KD-Klotho (n = 3). **H** The SA-β-Gal staining of HK-2 cells with KD-Klotho. **I** The content of MDA in HK-2 cells with KD-Kloth (n = 3). n = 3 biologically independent repeats. Two-sided Student’s t-test, data shown are mean ± SD. *P < 0.05, **P < 0.01, ***P < 0.001

Klotho is a promising target for future nephropathy treatments, addressing issues such as oxidative stress, mitochondrial abnormalities, and cell aging. Our previous research revealed that ABI3BP is up-regulated following irradiation-induced aging or UUO injury, coinciding with a down-regulation of Klotho. Importantly, ABI3BP knockout increased Klotho expression. GEO data from UUO also confirmed a significant decrease in Klotho expression (Fig. S5A), with a clear negative correlation observed between ABI3BP and Klotho (Fig. S5B). Thus, we hypothesized an interaction between ABI3BP and Klotho. To verify this hypothesis, we conducted a co-IP experiment, confirming the interaction between ABI3BP and Klotho (Fig. 6A, B).

To elucidate the effect of Klotho on aging and ferroptosis, we knocked out Klotho and carried out subsequent validation. KO-Klotho led to an increase in Fe^2+^ content in HK-2 cells (Fig. 6C), accompanied by elevated iron accumulation and ROS content (Fig. 6D). Furthermore, GPX4 and Nrf2 the expressions were down-regulated, while Acsl4 was significantly up-regulated (Fig. 6E). KD-Klotho significantly reduced Klotho mRNA levels, as confirmed by quantitative RT-PCR analysis (Fig. 6F). This manipulation caused a significant decrease in cell proliferation rate (Fig. 6G) and an increase in the percentage of senescent cells, identified by SA-β-gal staining (Fig. 6H). These observations suggest that Klotho deficiency promotes cellular senescence. Moreover, Klotho knockdown triggered a significant upregulation of MDA in HK-2 cells (Fig. 6I). MDA is a well-established marker of lipid peroxidation, a hallmark of ferroptosis. Taken together, these findings imply that Klotho deficiency can activate intracellular ferroptosis, thereby contributing to cellular senescence.

**Fig. 7:**
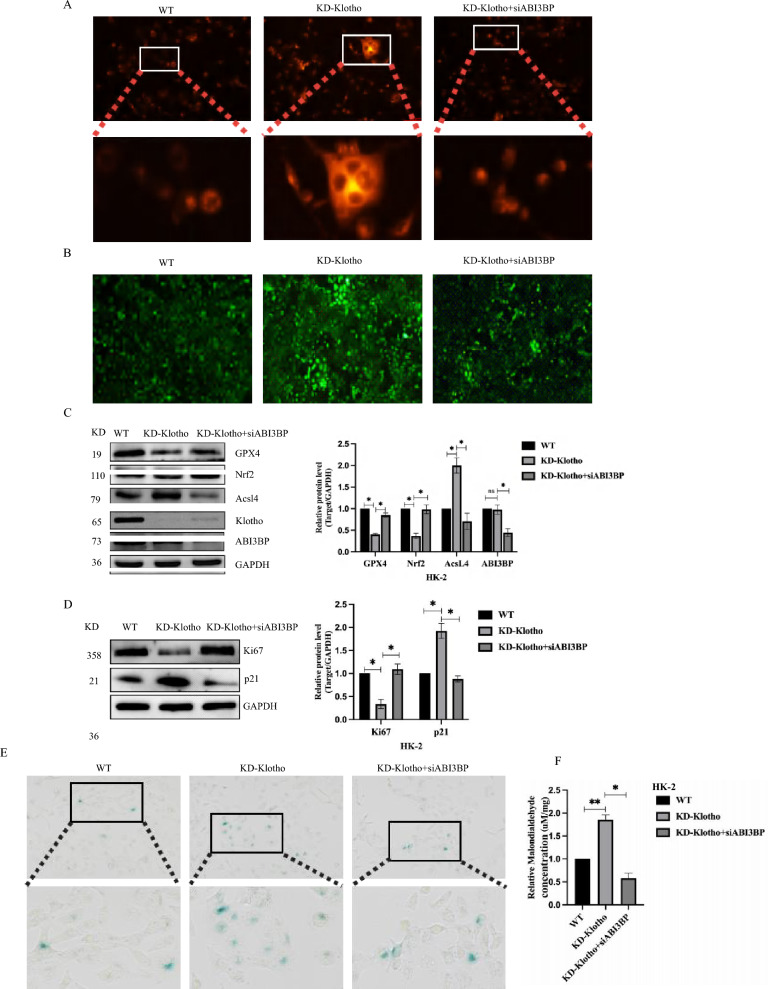
ABI3BP knock down alleviates cell senescence induced by of ferroptosis up-regulation caused by Klotho knock-down. **A** Ferrous ion fluorescent probe staining in HK-2 cells with si-ABI3BP (n = 3). **B** Increased ROS production in HK-2 cells with si-ABI3BP (n = 3). **C** Western Blot analysis of protein levels of Nrf2, GPX4, Klotho, and Acsl4 in HK-2 cells after si-ABI3BP (n = 3). **D** Western Blot analysis of protein levels of Ki67 and P21 in HK-2 cells (n = 3). **E** Detection of si-ABI3BP in HK-2 cells by SA-β-gal staining (n = 3). **F** si-ABI3BP reversed the upregulation of MDA induced by KD-Klotho in HK-2 cells (n = 3). n = 3 biologically independent repeats. Two-sided Student’s t-test, data shown are mean ± SD. *P < 0.05, **P < 0.01

To clarify the upstream and downstream relationship between ABI3BP and Klotho, we assessed ferroptosis levels with si-ABI3BP after KO-Klotho. Si-ABI3BP mitigated the increase in Fe^2+^ content in HK-2 cells caused by KO-Klotho (Fig. 7A) and decreased ROS content (Fig. 7B). The expression of key proteins GPX4 and Nrf2 recovered, while Acsl4 was significantly down-regulated (Fig. 7C). However, KO-Klotho did not alter the level of ABI3BP. These results confirmed the regulatory effect of ABI3BP on Klotho. The effect of the ABI3BP-Klotho-ferroptosis axis on aging was further validated. Western blot demonstrated that si-ABI3BP could reduce the increase in P21 caused by KO-Klotho and increase the expression of the proliferation-related protein Ki67 (Fig. 7D). The proportion of SA-β-gal positive senescent cells also significantly decreased after si-ABI3BP (Fig. 7E). These results also demonstrated that si-ABI3BP could reverse the upregulation of MDA induced by KD-Klotho (Fig. 7F). In summary, these findings suggest that inhibiting theABI3BP/Klotho/ferroptosis axis in renal tissues and cells by reducing ABI3BP levels could be considered a potential strategy to alleviate renal aging.

## Discussion

In the last century, human life expectancy has witnessed substantial growth, reaching an average of 76.5 years in developed countries and 65.4 years in developing nations [17]. Comprehensive population-based studies consistently demonstrate prevalent impaired renal function among the elderly. Renal aging is characterized by a progressive decline in renal tubular dysfunction, reduced sodium reabsorption, increased potassium excretion, and decreased urine concentration, contributing to increased susceptibility to acute kidney injury (AKI) [18]. Structural changes, such as decreased nephron size and number, tubulointerstitial, thickening of the glomerular basement membrane, and exacerbated glomerulosclerosis, further underscore the complexities of renal aging [19, 20].

ABI3BP, also referred to as Tarsh, is located on chromosome 3q12.2 in humans, and is recognized as an extracellular/interstitial matrix protein pivotal in cell–matrix adhesion. Although expressed in various tissues throughout the body, including the brain [21], its role in kidney diseases remained unexplored until our study. Our investigation reveals a substantial increase in ABI3BP expression in kidney tissues and renal tubular epithelial cells of aging mice induced by radiation. Utilizing the GEO database and the UUO model, we observed significantly elevated ABI3BP expression in renal tissues with injury compared to the control group. The expression of ABI3BP showed a progressive increase with the extension of the operation time. Interfering with ABI3BP expression demonstrated a potential to alleviate aging in renal tubular epithelial cells induced by irradiation and UUO, underscoring the involvement of ABI3BP in renal aging and suggesting that interfering with its expression could potentially delay the aging process. In addition, studies have highlighted the presence of ABI3BP in normal lung tissues of mice, correlating with the aging of both mouse embryonic fibroblasts (MEFs) and lung tissues [22, 23]. This association exhibits age-dependent patterns. Notably, a significant decrease in ABI3BP expression is observed in the majority of malignant thyroid tumors. The re-expression of ABI3BP in nude mice tumor model results in the inhibition of cell growth, activity, migration, and overall tumor growth. This phenomenon is linked to the induction of cell senescence through the activation of the p21 signaling pathway [14]. These findings align with our conclusions, suggesting that ABI3BP may serve as an aging-related factor.

Ferroptosis recognized as a non-regulatory form of cell death characterized by iron dependence and excessive lipid peroxides [24], has gained prominence since its conceptualization in 2012. Although the detailed mechanism of ferroptosis remains incompletely elucidated, accumulating evidence establishes its intricate links with various diseases. Aging, an irreversible physiological process and a key pathogenic factor in aging-related diseases encompasses diverse mechanisms, with ferroptosis as one of them [25]. Existing literature has reported increased occurrences of ferroptosis with age in organs such as the kidneys, spleen, liver, ovary, uterus, cerebellum, and bone marrow, associated with iron accumulation [26]. In this study, increased levels of iron ions and ROS were evident in kidney tissues and HK2 cells of aging mice, accompanied by a significant decrease in the key ferroptosis indicators, GPX4 and Nrf2. This implies that ABI3BP could potentially promote renal aging by regulating ferroptosis.

Klotho, an anti-aging gene discovered in 1997 [27], serves a dual role by functioning as a renal protective agent [28–30]. Studies have reported a correlation between changes in Klotho levels, the degree of renal function damage, and the prognosis of chronic kidney disease (CKD) [28, 31]. In this study, we observed a significant decrease in Klotho expression in the kidney tissue of the experimental group compared to the control group. Importantly, a significant negative correlation emerged between ABI3BP expression and klotho levels. Further examination revealed that knocking down ABI3BP led to an elevation in Klotho expression, while overexpressing ABI3BP resulted in its reduction. Immunoprecipitation confirmed a direct interaction between ABI3BP and Klotho. These findings strongly suggest that ABI3BP promotes the senescence of renal tubular epithelial cells by inhibiting Klotho expression. Furthermore, inhibiting Klotho expression led to increased levels of iron ions and ROS in HK2 cells, accompanied by a down-regulation of GPX4 and Nrf2, ultimately promoting cell aging. Notably, interference with Klotho also inhibited the reduction in ferroptosis caused by ABI3BP expression. These results indicate that ABI3BP promotes ferroptosis by inhibiting Klotho expression, thereby contributing to cell senescence. Recently, Xiong et al. [32] reported that Klotho could protect aged cardiomyocytes by reducing ferroptosis, aligning with our study’s results.

In conclusion, this study identifies an upregulation of ABI3BP in aged renal tubular epithelial cells, which further promotes ferroptosis and induces renal aging by inhibiting Klotho expression.

## Data Availability

All the data used during the study are available from the corresponding author on request.
